# Bullying Victimization as a Risk Factor for Gun Carrying Among US Adolescents

**DOI:** 10.7759/cureus.31785

**Published:** 2022-11-22

**Authors:** Rawlica Sumner, Marc Ganz, Menachem Jacobs, Christopher Alessandro, Daniel Fuchs, Steven Gamss, Daniel Miller

**Affiliations:** 1 Public Health Sciences, State University of New York Downstate Health Sciences University, New York City, USA; 2 Internal Medicine, Coney Island Hospital, New York City, USA; 3 Medicine, Northwell Health, New York City, USA; 4 Internal Medicine, Icahn School of Medicine at Mount Sinai, Queens Hospital Center, New York City, USA

**Keywords:** school shootings, gun carrying, bullying, public health education, public health and safety

## Abstract

Schools are a place for learning and education, and in a learning environment, everyone should feel safe. Gun violence is a serious concern for students and teachers alike in a school setting. There may be a multifaceted explanation for why a student would be carrying a firearm or any other weapon in school. Our research explores the association between being a victim of bullying and carrying a firearm. The national Youth Risk Behavior Surveillance System survey was utilized to assess the increased incidents of bully victims carrying firearms. Several efforts, including the passing of laws, have been made to prevent the carrying of firearms. However, additional attempts must be made to reduce bullying in schools as well, so that students do not feel the need to carry a weapon, and schools can be a safe environment for everyone.

## Introduction

Bullying can pose a key threat to the safety of a given school environment. In the 2015 Youth Risk Behavior Survey (YRBS), 6% of the students who responded were found to be threatened with a weapon on school property at least once over the course of the past 12 months [1]. The Gun-Free School Zones Act (GFSZA) prohibits the possession of a gun within school zones, but this law does not apply to individuals licensed by the state to possess a gun [2]. Additionally, the Gun-Free Schools Act (GFSA) mandates that states that receive certain federal funds must suspend students for at least one year if a firearm is brought to a school or is kept within a school [2]. However, while the GFSZA and GFSA are both federal laws, in some states, K-12 schools can permit students in possession of a permit to carry a gun [2,3]. Many studies have shown an association between being the victim of bullying and carrying a weapon, particularly a firearm [4-7]. The different manifestations of bullying have varying likelihoods of carrying firearms [4-6]. It is also evident that differences in victim classes (non-victims, those who experience different types of bullying, and those who are highly victimized) may not be good predictors of the likelihood to carry a firearm [6]. Nevertheless, there is evidence that repeated bullying and highly victimized populations have a higher prevalence of carrying a gun. It has also been suggested that bullying is not necessarily the main risk factor for carrying a gun [6,7].

As it is important that schools maintain a safe environment for teaching, it is crucial to understand the role of bullying victimization as a risk factor for adolescents to carry a firearm. We believe that bully victimization on school property may increase the risk of an adolescent carrying a firearm, and may further instigate violence on and off school property. This study seeks to evaluate whether adolescents who have been bullied in the past 12 months on school property are more likely to carry a gun than those who have not been bullied in the past 12 months.

## Materials and methods

Data

We used the 2019 national Youth Risk Behavior Surveillance System (YRBSS) survey as our source of data for this study. The Physicians Journal of Medicine Institutional Review Board reviewed and approved this study. The YRBSS monitors the health risk behaviors of youth and adults in the United States. These risk behaviors are thought to contribute to social issues, leading to causes of death, and disability among the population. In addition to monitoring the prevalence of chronic diseases such as obesity and asthma, the YRBSS also monitors other behaviors including but not limited to tobacco use, inadequate physical activity, alcohol and drug use, and unhealthy dietary behaviors. Surveys gathered by YRBSS are representative of students in private and public schools from the 9th to 12th grades. The total sample size of this study was 13,447. The mean age was approximately 16 years, with the age ranging from 12 to 18 years.

Measures

The outcome variable we used was carrying a gun at least once during the past 12 months (not including days where a gun was only carried for hunting or for sport, such as target shooting). Reports of carrying a gun for one day, two or three days, four or five days, and six or more days were coded as Yes, and an answer of 0 days was coded for as No. The predictor variable used in this study was bullying victimization. This variable was measured by whether the participant answered Yes or No to the question which asked whether the student was bullied on school property during the past 12 months. Demographic covariates such as gender, age, grade, and race were analyzed to help understand whether different demographics are significantly associated with carrying a gun.

Statistical analysis

The chi-square test was used to measure the association between the predictor and outcome variable. A p-value of less than 0.05 was considered to be statistically significant at the 95% confidence level. Multivariate logistic regression was used to analyze the relationship between the predictor and outcome variable as well as covariates. Both crude and adjusted odds ratios (ORs) along with their confidence intervals were computed. All analyses were performed using RStudio (Posit, Boston, MA).

## Results

Among bullied students, 5.9% carried a gun and among non-bullied students, 4% carried a gun. Approximately 501 (4.4%) students reported carrying a gun within the past 12 months while 2703 (19.8%) students reported being bullied on school property within the past 12 months. The number of female and male participants was comparable. Being male was significantly positively associated with being bullied, at p<0.001. Grade level and age were also significantly positively associated with being bullied at p<0.001. While the Black, White, Asian, and Hispanic race/ethnicity groups were significantly associated with being bullied (at p<0.001), the Other category (comprising American Indian/Alaska Native and Native Hawaiian/Other Pacific Islander) was found to be significantly associated with being bullied at p=0.01 (Tables 1, 2).

**Table 1:** Characteristics of 2019 YRBSS participants M = mean, SD = standard deviation, YRBSS = Youth Risk Behavior Surveillance System

Variable	Bullied in the past 12 months on school property (%)	Not bullied in the past 12 months on school property (%)	p-value
Total	2703 (19.8)	10,744 (78.6)	
Carrying a gun	123 (5.9)	349 (4.0)	
Gender			
Male	1027 (38.6)	5496 (51.5)	<0.001
Female	1634 (61.4)	5169 (48.5)	
Grade, M (SD)	10.3 (1.1)	10.4 (1.1)	<0.001
Age, M (SD)	15.8 (1.3)	16.0 (1.2)	<0.001
Race/ethnicity			
Black	275 (10.5)	1711 (16.4)	<0.001
White	1578 (60.5)	5028 (48.1)	<0.001
Asian	68 (2.6)	541 (5.2)	<0.001
Hispanic	108 (4.1)	890 (8.5)	<0.001
Other	56 (2.1)	152 (1.5)	0.01

**Table 2:** Bullying victimization and carrying a gun

	Adjusted odds ratio	95% CI
Bullied	1.83	1.45-2.31
Male	4.70	3.72-6.01
Grade	1.05	0.89-1.25
Age	1.04	0.89-1.21
Race/ethnicity		
Black	1.34	1.02-1.77
White	0.60	0.47-0.76
Asian	0.28	0.13-0.55
Hispanic	0.80	0.54-1.17
Other	0.81	0.34-1.68

There was a statistical significance between carrying a gun and bullying victimization at a p-value of <0.001. Adolescents who were bullied on school property within the past 12 months had 49% higher odds of carrying a gun than adolescents who were not bullied on school property within the past 12 months. When adjusted for gender, grade level, age, and race/ethnicity, adolescents who were bullied on school property within the past 12 months had 83% higher odds of carrying a gun than adolescents who were not bullied on school property within the past 12 months. Being a male or being Black had significantly higher odds of carrying a gun. This contrasts with being White or Asian that revealed significantly lower odds of gun carrying among adolescents (Table 2).

## Discussion

This study primarily sought to determine the association between adolescents who had been bullied on school property within the past 12 months and carrying a gun. We found that there was a significant association between being bullied and carrying a gun even when adjusted for demographic covariates such as age, gender, grade level, and race/ethnicity. Secondarily, we found that being Black and being male increased the likelihood of carrying a gun.

Our primary finding is consistent with the current literature that shows that being bullied has been associated with greater odds of carrying a weapon in general (although it is not specific as to whether the weapon carried was a gun) [8]. While our study failed to measure whether students carried guns on school property, it is important to consider the possibility that respondents who did carry a gun, potentially did so on school property. While bullying does increase the likelihood of carrying a gun to school, so do symptoms of anxiety and depression among Black males [9]. Another factor that is associated with gun carrying among adolescents is parental disengagement (factors such as poor parental involvement and poor parent-son relationship quality) during childhood [10]. Our study also found that there was a significant association between grade level and gun carrying, which is also consistent with the literature. It has been noted that as the grade level increased, the odds of having carried a gun decreased (in the comparison of 9th to 11th graders) [11].

Our study did not investigate the reason as to why the bullied would carry a gun. Some possible theories include self-defense or even revenge. In the former theory, it is important to recognize that carrying a gun itself does not seem to correlate with a decrease in the probability of getting shot at in the case of an assault [12]. Furthermore, another study demonstrated a positive correlation between suicide rates in minors and gun ownership in a home [13]. This study demonstrated the need for further investigations as to the root cause of gun carrying in this population type as gun carrying may cause more harm than good.

In the future, other covariates such as perceived safety should be analyzed. A limitation of this study is that the YRBSS did not specify what type of bullying was experienced by those who were bullied (e.g. physical, social, etc.). Understanding whether there are significant differences in the type of bullying experienced and gun carrying will be useful to further decide where the focus should be for potential targeted interventions and policies for the future. Response bias is also a limitation of this study as some students who were bullied or who did carry a gun may have not wanted to disclose that information. Another limitation is that the YRBS data is cross-sectional. Thus, while associations can be made, causal relationships cannot be established. Nevertheless, a strength of this study is that it utilized a large sample size. Since the sample size is representative of adolescents from grades 9 through 12, this study can be generalized to the US population of adolescents in both public and private schools.

## Conclusions

Our study findings that show that bully victimization is associated with gun carrying can aid in making a case for bullying prevention interventions. In addition to bullying, other contributory factors to gun carrying should be studied. More research is needed to understand whether guns are being carried on school property by those who have been bullied, and also, the implications of doing so to create policies that will maintain a safe learning environment for students.
